# Supplementary material for: Effect of Vedolizumab on Anemia of Chronic Disease in Patients with Inflammatory Bowel Diseases
Source: J Clin Med. 2020 Jul 6;9(7):2126. doi: 10.3390/jcm9072126 (PMC7408734; doi:10.3390/jcm9072126)
Supplement: Supplementary file 1 [file jcm-09-02126-s001.pdf]

Supplementary Materials

Table S1. Clinical and demographic characteristics of the patients.

Variable	IBD Patients (*n* = 75)
median age, years (IQR)	49 (18–77)
median disease duration, years (IQR)	13 (1–44)
male gender, *n* (%)	36 (48%)
smoking status, *n* (%)	
never	36 (48%)
former	30 (40%)
current	9 (12%)
CD, *n* (%)	25 (33%)
Montreal disease location, *n* (%)	
L1 (ileal disease)	11 (33%)
L2 (colonic disease)	2 (6%)
L3 (ileocolic disease)	20 (61%)
Montreal disease behavior, *n* (%)	
B1 (non-structuring, non-penetrating)	10 (30%)
B2 (structuring)	9 (12%)
B3 (penetrating)	14 (58%)
perianal disease, *n* (%)	8 (24%)
prior surgery, *n* (%)	16 (48%)
UC, *n* (%)	50 (67%)
E1 (proctitis)	3 (6%)
E2 (left-sided colitis)	15 (30%)
E3 (extensive colitis)	32 (64%)
prior TNF antagonists, *n* (%)	51 (68%)

IQR: Interquartile range. CD: Crohn′s disease. UC: ulcerative colitis. TNF: Tumor Necrosis Factor.

Table S2. Distribution of baseline demographic/clinical characteristics and clinical response to Vedolizumab in patients with pure ACD and patients with mixed type anemia.

Variable	Patients with Pure ACD (15/35)	Patients with Mixed Type Anemia (20/35)	*p* Value
Male gender	7 (47%)	8 (40%)	*p*=0.693
Age < 65 years	13 (87%)	18 (90%)	*p*=0.759
Crohn's disease	5 (33%)	6 (30%)	*p*=0.833
Ulcerative colitis	10 (67%)	14 (70%)	*p*=0.833
Current smokers	2 (13%)	1 (5%)	*p*=0.383
Previous anti-TNF	9 (60%)	17 (85%)	*p*=0.094
Concomitant steroids	6 (40%)	12 (60%)	*p*=0.241
Concomitant immunosuppressors	0	1 (5%)	*p*=1
Hemoglobin (median, IQR) (gr/Dl)	11.4 (8.3–12.5)	10.7 (8.7–12.7)	*p*=0.064
Severe clinical activity	0	3 (15%)	*p*=0.243
Moderate clinical activity	11 (73%)	12 (60%)	*p*=0.410
Mild clinical activity	4 (27%)	5 (25%)	*p*=0.911
Severe endoscopic activity *	11 (73%)	10 (59%)	*p*=0.388
Moderate endoscopic activity	3 (20%)	5 (29%)	*p*=0.539
Mild endoscopic activity	1 (7%)	2 (12%)	*p*=0.621
CRP > 5 mg/L	10 (67%)	13 (65%)	*p*=0.918
CRP value (median, IQR) (mg/L)	6 (3.2-28)	23 (3.8-56.3)	*p*=0.317
Anemia improvement	7 (47%)	6 (30%)	*p*=0.312
Anemia resolution	4 (27%)	7 (35%)	*p*=0.599
IBD clinical response to Vedolizumab at week 14	9 (60%)	10 (50%)	*p*=0.556
IBD clinical response to Vedolizumab at week 24	9 (60%)	10 (50%)	*p*=0.556

ACD: Anemia of Chronic Disease. Anti-TNF: anti-Tumor Necrosis Factor. CRP: C reactive protein. IBD: Inflammatory Bowel Disease. * Endoscopic data available in 15/15 patients with pure ACD and in 17/20 patients with mixed type anemia.
